# Smart packet prioritization in cognitive radio networks for smart agriculture

**DOI:** 10.1038/s41598-026-62682-1

**Published:** 2026-07-23

**Authors:** Enas Selem, Abeer Alattal, Abdel Hamid A. Shaalan, Nirmin M. Abdelwahab

**Affiliations:** 1 Information Technology department, Faculty of Computers and Information, Suez University, Suez, Egypt; 2 Electronics and Communication Department, Faculty of Engineering, Zagazig University, Zagazig, Egypt

**Keywords:** Ambient smart agriculture, Cognitive radio networks, Deep reinforcement learning, Dueling double DQN (D3QN), Dynamic aging threshold, Priority queuing management, Engineering, Mathematics and computing

## Abstract

The quick evolution in the field of Smart Agriculture, along with the Internet of Things (IoT), has created the need for developing wireless communication paradigms with the ability to handle heterogeneous data for different applications with different degrees of priority. This paper proposes an autonomous packet priority management framework for IEEE 802.11af-based CRNs to meet the heterogeneous data demands of Smart Agriculture and IoT. By employing a Dueling Double Deep Q-Network (D3QN) with a new dynamic aging threshold, the model avoids data starvation while ensuring reliable transmission of high-priority agricultural data over TVWS. Simulation results demonstrate that the D3QN framework achieves significantly better performance compared to standard and DQN-based models, especially in highly congested conditions (λ > 8). The proposed scheme reduces average delay for high-priority packets by 16.1% compared to the Baseline and by 10.5% compared to standard DQN under high-load conditions, and achieves approximately 14.8% reduction in relative energy consumption per high-priority packet, while maintaining comparable throughput. These results demonstrate a more robust and energy-efficient solution for real-time intelligent farming environments.

## Introduction

### The revolution of smart agriculture

The global agri-landscape is currently in a state of transformation and is referred to as Agriculture 4.0. This refers to a revolution driven by the need to enhance food production efficiency to feed and sustain a growing global populace while remaining mindful of environmental responsibility. At the heart of this revolution is the IoT^1^, which primarily focuses on ensuring seamless sensing, communication, and actuation within the agri-ecosystem. Smart Agriculture is no longer a luxury but a necessity, driven by a reliance on a number of Wireless Sensor Devices (WSDs), which are tasked with ensuring sensing and monitoring of soil moisture, temperature, crop conditions, and environmental threats. Cognitive Radio Networks (CRNs) act as an enabling technology to allow agricultural sensors to access vacant TV White Space spectrum bands in an opportunistic manner to cater to the spectral flexibility requirements^2,3^.

### Communication challenges in rural areas

In spite of the many opportunities offered by IoT technologies, the implementation of smart farms, in practice, encounters significant problems in the provision of an adequate communication infrastructure, especially in rural and countryside areas. Wi-Fi technology or cellular networks may not perform adequately because of the high costs and insufficient coverage. Similarly, there may also be problems with significant degradation of network signals while traversing a terrain with high foliage. However, this problem can be mitigated by the emergence of a new technology standard, designated as “IEEE 802.11af,” which employs Television White Spaces frequency bands for its operations. Since it supports the Sub-1 GHz frequency band, it has better propagation capabilities, by which it can transmit signals across a distance.

### Data priority and heterogeneous traffic

Various smart agriculture applications include data packets with varying levels of priority and urgency. Such data can generally be categorized into three distinct classes^4^:

1. Critical Emergency Data (Red Packets): Such as fire alerts, sudden equipment failure, or irrigation pipe bursts, requiring near-instantaneous transmission.
2. Routine Monitoring Data (Yellow Packets): Such as periodic soil and crop health updates that are important but can tolerate moderate delays.
3. Logistical and Background Data (Green Packets): Such as non-essential telemetry or software updates.

The “Humanized Computing” part of the network refers to the network’s capability to differentiate between these classes and favor the safety of human life and crop growth. However, conventional Medium Access Control (MAC) procedures of the 802.11af rely on the interpretation that all packets are largely similar, causing “congestion collapse” when data traffic is heavy. Much of an urgent data “emergency call” can be held up by “routine” background data calls in the event of a local disaster, such as fire.

### Challenges of conventional resource management

In IEEE 802.11af CRNs, conventional prioritization procedures can face challenges under congestion. Rigid prioritization may lead to starvation of low-priority traffic, while adaptive methods advance packets based on the longest wait times, even so it often depends on tuned parameters that may not generalize well under varying wireless conditions. In addition, the nature of rural environments including variable interference and fluctuating traffic intensity makes static or rule-based approaches less effective, motivating the need for more intelligent and self-adaptive solutions.

### The emergence of deep reinforcement learning (DRL)

To solve these problems, researchers are inclined towards the incorporation of Artificial Intelligence techniques, particularly Deep Reinforcement Learning (DRL). DRL enables the design of an intelligent agent that learns optimal decisions through interaction with its environment^5^. In the case of IEEE 802.11af networks, DRL agent observes queue states and offered traffic load (λ) and adapts the aging threshold accordingly. However, in the case of Reinforcement Learning-based algorithms, such as Deep Q-Network (DQN)^6^, it was observed that the “overestimation bias” of taking optimal decisions may lead to unstable behavior in critical environments, like smart farms.

### Proposed solution: the D3QN framework

This paper proposes an advanced framework for resource management that is based on the Dueling Double Deep Q-Networks (D3QN). D3QN is designed to mitigate key limitations of standard DQN^7,8^. In the D3QN architecture, the state-value estimation is decoupled from the advantage function estimation, while the use of the Double action evaluation enables the agent to be stable in decision-making, which is crucial for the mission-critical nature of the agricultural IoT. The essential element of our contribution is the intelligent adaptation of the aging threshold. While a static system only acknowledges the “cost” associated with delayed delivery for different classes of packets and learns a policy to minimize a weighted cost function, our D3QN agent behaves differently by dynamically adapts the aging threshold to prioritize urgent information while preventing starvation of non-urgent traffic, supporting what we call “humanized computing.”

### Research objectives and paper organization

The main objective of the present study is to show that an autonomous agent resulting from a D3QN-based approach is able to highly increase Quality of Service (QoS) and energy efficiency in IEEE 802.11af-based systems, following a rigorous performance evaluation against reference fixed-threshold scheduling schemes and standard DQN-based ones.

#### Research gap

While the body of research on using Deep Reinforcement Learning for wireless resource management is fast growing, a critical review of the literature shows that several challenges remain and will be addressed in this work:

1. Static vs. Dynamic Priority Mismatch: Most of the current research on IEEE 802.11af focuses on physical layer optimization or basic spectrum sensing. There is a notable lack of research in high-layer traffic management that can autonomously cope with the extreme heterogeneity of Smart Agriculture data where, most of the time, urgency of an emergency alert is lost in static queuing models.
2. Limitations of Traditional DQN in Wireless Environments: Standard DQN has been applied to networking, which often faces overestimation bias and instability with high dynamics, particularly in scenarios such as TVWS. Most of the existing works have not considered more robust architectures, such as Dueling Double DQN, which is able to separate state-value estimation from action advantages in more smooth convergence.
3. The paper finds a huge gap in the “Aging Threshold” dynamic optimization. Most of these adaptive systems use rigid, rule-based functions that are often unable to adapt to nonlinear traffic surges. There is a lack of “humanized” computing frameworks that can balance the rigid latency requirements of emergency data with the need to prevent “packet starvation” for routine monitoring sensors.
4. Energy-Efficiency Integration: Past literature lacks an explicit association of DRL-based scheduling with the relative battery life of constrained IoT devices in a multi-class priority scenario. Moreover, it appears that the “synergistic effect” of intelligent D3QN-based scheduling of packets combined with minimized waiting time with usage of IEEE 802.11af is still unexplored.

In this context, the present research fills the above-identified gaps and offers an innovative, independent, and energy-conscious framework, transforming IEEE 802.11af from an opportunistic medium of transmission to an intelligent and context-sensitive medium of communication.

Beyond the agricultural-CRN literature reviewed above, the trade-off between timely delivery of high-priority data and overall energy efficiency is a recurring challenge across other resource-constrained, heterogeneous wireless IoT deployments. For instance, Mehmood et al.^9^ proposed a QoS-based multi-path routing scheme for wireless body area networks (WBANs) that balances delay-sensitive health data against energy budgets, while Mehmood et al.^10^ designed a mobile agent-based data aggregation scheme to reduce energy consumption in WBANs. These works reinforce the broader motivation for autonomous, learning-based scheduling, here specialized for the distinct traffic patterns and opportunistic spectrum-access characteristics of IEEE 802.11af-based smart agriculture, which neither the WBAN-focused studies nor the agricultural CRN works discussed earlier address jointly.

#### Contributions

The main contributions of the current paper can be listed as follows:

1. Development of a D3QN-Based Autonomous Framework: In this paper, we are proposing an efficient, autonomous framework based on the D3QN algorithm to carry out improved resource management in IEEE 802.11af wireless networks. By exploiting the dueling structure for separating state-value and advantage functions, this framework ensures high accuracy of the action selection in dynamic Cognitive Radio environments. This would enhance network throughput by intelligently managing heterogeneous traffic flows and ensure that spectrum resources of TV White Spaces are utilized to the maximum without compromising on operational stability under heavy load conditions.
2. Dynamic Aging Threshold for Starvation Mitigation: The core novelty of the proposed framework will be the autonomous dynamic aging threshold adjustment, as this is designed to resolve the starvation problems commonly experienced by low-priority normal agricultural data due to the dominance of emergency transmissions. By adaptively tuning the threshold, the system ensures that the application’s strict latency requirements for high-priority emergency packets are met, while not indefinitely delaying normal data packets. This balanced prioritization ensures a fair distribution of resources to keep the flow of normal, routine monitoring data running together with critical alerts in smart farming environments.
3. Mitigation of Overestimation Bias: The integration of the Dueling architecture and Double Q-Learning in the proposed system mitigates the issue of Overestimation Bias due to the separation of state value and action advantages, which improves the stability of the system compared to traditional DQN systems.
4. Energy-Efficient Priority Management: We propose a dynamic priority-shifting mechanism, which minimizes active waiting time of sensor nodes. This leads to a measurable reduction in relative energy consumption, which is very important for the longevity of the battery-powered IoT devices in smart farming.
5. Comprehensive Performance Validation: We conduct extensive comparative analysis by benchmarking the proposed D3QN agent against Baseline, Adaptive, and standard DQN models. The results show that D3QN maintains lower latency and higher stability, especially under high traffic loads, which is a common scenario in emergency agricultural monitoring.

#### Paper organization

The rest of this work is organized as follows: In  “Related works” section outlines the related works about both TVWS and DRL. Then, System model and methodology  section illustrates the system model and introduces the methodology of D3QN. In  “Simulation results and discussion” section demonstrates the simulation results and gives a detailed discussion about the delay-throughput-energy tradeoffs. Finally, “Conclusion” section concludes the paper and in “Future research directions” section suggests future research directions.

## Related works

Recent advancements in the international journals like Journal of Ambient Intelligence and Humanized Computing (JAIHC) and related IEEE/Springer literature have showcased the transformative potential of Deep Reinforcement Learning (DRL) in managing complex IoT environments.

- **DRL for Resource Management and QoS**:

The challenge of resource allocation in dynamic networks has been a focal point of recent studies. For instance, Liu et al.^11^ proposed a DRL-based framework for task offloading and resource allocation in IoT networks, demonstrating that deep learning can effectively adapt to time-varying traffic. Similarly, Zhang et al.^12^ investigated the use of standard DQN for channel sensing in Cognitive Radio Networks (CRN), though their model primarily focused on throughput without deeply addressing the latency requirements of heterogeneous data types. Our work extends these concepts by employing a **D3QN** architecture, which offers better stability and faster convergence in multi-priority smart agriculture settings.

in^13^, the authors presented a dynamic spectrum access solution in TVWS in rural areas based on standard Deep-Q Network (DQN) architecture. The proposed solution performed well in identifying free channels of maximum bandwidth but used a standard single stream DQN, which is naturally vulnerable to overestimation bias, resulting in suboptimal channel allocation in highly congested conditions. Moreover, in the reference paper, the proposed solution treated all IoT traffic as homogeneous, which is highly undesirable in a scenario where IoT traffic from smart farms needs to be prioritized due to the urgency of alert messages. In contradiction, in our proposed D3QN, we use a Dueling network, which is necessary for providing robust results in conditions of heterogeneous data with different levels of urgency.

Another area of primary focus remains energy efficiency, which was examined in the research work of Khalid and Razzaq in their article entitled “Heuristic Adaptive Algorithms for Scheduling in IEEE 802.11af.”^14^. Although this method was successful in achieving short-term results, these models follow pre-defined mathematical rules that cannot scale with given unpredictable bursts of traffic. Unlike heuristic models, our approach is autonomous and self-learning, allowing adaptive efficiency improvements through real-time adaptation without requiring human interventions in parameter tuning.

While it is proposed by Nguyen et al.^15^ that, for IoT network interference mitigation, the Multi-Agent RL technique may be leveraged, it may be impractical for IoT devices because of the associated computational cost and communication latency involved. Our proposed system leverages D3QN, a computationally efficient single-agent variant at the Access Point, to ensure the optimization of the entire cluster without affecting the end device batteries. then, we will explore the work in Cognitive Radio and IEEE 802.11af in Smart Environments.

- **Cognitive Radio and IEEE 802.11af in Smart Environments**:

The utilization of TV White Spaces (TVWS) via the IEEE 802.11af standard has been recognized as a key enabler for long-range agricultural IoT. Wang and Smith^16^ explored the performance of 802.11af in rural areas, highlighting that while it provides excellent coverage, it suffers from packet collisions during high-traffic scenarios. To mitigate this, Kumar et al.^17^(published in JAIHC) suggested an adaptive medium access control (MAC) protocol. However, their approach relied on static priority rules, which often lead to the starvation of low-priority monitoring data. This gap in the literature justifies our proposal for a dynamic aging threshold that autonomously balances emergency and routine data.

Further improvements in stability have been proposed in the work of Ahmed et al.^18^ using Double DQN in spectrum sensing. Although DDQN partly fixes the overestimation problem, it fails to effectively tackle the exploration-exploitation balance, hence slow convergence. Our method incorporates the Dueling algorithm and DDQN, thereby speeding up convergence and improving response to emergency packets. Under the context of MAC optimization, Li and Kim^19^ adjusted the Contention Window (CW), but their algorithm failed to tap into the potential of the aging threshold within the queue. Our algorithm, by contrast, specifically tackles the “Head-of-Line” blocking difficulty by dynamically optimizing the aging threshold, thereby preventing low-priority packets from being indefinitely delayed behind congested high-priority traffic.

Another similar work done by Zhang et al., as published in^20^, has used the concept of Reinforcement Learning to optimize the levels of latency in any given IoT network. However, the work **primarily** discussed maximizing the levels of throughput and has not taken into consideration the propagation properties that are unique to the Sub-1 GHz TVWS band, as studied in this paper. Consequently, the focus has shifted from channel availability alone to traffic-aware scheduling mechanisms that explicitly address starvation and fairness in agricultural IoT.”

- **Intelligent Agriculture and Starvation Mitigation**:

In the context of humanized computing for smart farming, Ahmed et al.^21^ emphasized the need for “Fairness-aware” scheduling. They argued that in intelligent farming, while emergency alerts (e.g., fire or irrigation failure) require immediate transmission, the starvation of periodic soil moisture data can lead to inaccurate long-term analytics. While their study used fuzzy logic for thresholding, our framework utilizes the Dueling architecture of D3QN to learn the optimal advantage of each threshold adjustment action, providing a more robust and autonomous solution for real-time agricultural monitoring.

Alternative methodologies, such as the fuzzy logic-based system for irrigation proposed by Mohanty et al.^22^, require in-depth knowledge to formulate rigid rules that respond to non-linear dynamics of wireless fading channels. Our proposed DRL-based solution, being model-free, leverages DRL and optimizes policies based only on the network data, hence being more robust compared to conventional solutions: it can handle rural environment fluctuations well.

Moreover, in a very recent work by Demirtürk and Bayrakdar^23^, the concept of cognitive sensor network was proposed in the context of emergency notifications for smart agriculture. In this work, cognitive radio is used in an effective manner for improving reliability in emergency situations. Although this work is focused on the notification mechanism in cognitive communication, it is not fully effective in handling the buffer management, ‘Head-of-Line’ blocking problems, etc. Our research builds on this direction by integrating D3QN to optimize the dynamic aging threshold at the MAC-layer queues, thereby prioritizing emergency packets while mitigating starvation for routine data, achieving a 16.1% reduction in average delay for high-priority packets under high-load conditions.

Another recent paradigm shift in agricultural monitoring involves the use of mobile sinks to enhance energy efficiency. Fouad et al.^24^ proposed the use of weather-aware drone sinks in achieving reliable communications in the presence of harsh environmental conditions. Although the authors’ proposed work is highly beneficial in the optimization of the physical layer in data collection, the essence of the proposed work still lies in the movements of the sink node itself. Thus, in our paper, the opposite problem is addressed, which is the management of the MAC layer’s queue once the reliable connection is established through the drone/AP itself, where improper packet scheduling without D3QN would otherwise result in data loss (Tables 1 and 2).

**Table 1:** Summary of related works.

Ref.	Author & Year	Methodology	Key Contribution / Focus	Limitations / Research Gap
^11^	Liu et al. (2022)	DRL Framework	Task offloading and resource allocation in IoT.	Did not prioritize heterogeneous data types.
^12^	Zhang et al. (2021)	Standard DQN	Channel sensing in Cognitive Radio Networks (CRN).	Focused on throughput; ignored latency for mixed traffic.
^13^	He et al. (2022)	Single-stream DQN	Dynamic spectrum access in TVWS for rural areas.	Vulnerable to overestimation bias; treated traffic as homogeneous.
^14^	Khalid & Razzaq (2023)	Heuristic Algorithms	Scheduling for energy efficiency in IEEE 802.11af.	Static rules; cannot scale with unpredictable traffic bursts.
^15^	Nguyen et al. (2024)	Multi-Agent RL	Interference mitigation in IoT networks.	High computational cost and latency for IoT devices.
^16^	Wang & Smith (2020)	Performance Analysis	Coverage analysis of 802.11af in rural areas.	Identified packet collisions but provided no MAC-layer solution.
^17^	Kumar et al. (2023)	Adaptive MAC	Traffic management in smart environments.	Relied on static priority rules, leading to data starvation.
^18^	Ahmed et al. (2023)	Double DQN (DDQN)	Stable spectrum sensing in cognitive radio.	Slow convergence due to exploration-exploitation imbalance.
^19^	Li & Kim (2022)	DQN-based CW	Contention Window (CW) adjustment in WLANs.	Failed to optimize the aging threshold within queues.
^20^	Zhang et al. (2024)	Reinforcement Learning	Latency optimization for IoT traffic.	Ignored Sub-1 GHz propagation properties unique to TVWS.
^19^	Ahmed et al. (2021)	Fuzzy Logic	Fairness-aware scheduling in agricultural IoT.	Lacked autonomous learning for real-time monitoring.
^22^	Mohanty et al. (2021)	Fuzzy Logic	Resource management for irrigation.	Required rigid expert rules; less robust to fading channels.
^23^	Demirtürk & Bayrakdar (2025)	Cognitive Sensors	Emergency notifications in smart agriculture.	Did not address buffer management or Head-of-Line blocking.
^24^	Fouad et al. (2025)	Weather-aware Drones	Reliable communications via mobile sinks.	Focused on physical layer/mobility; ignored MAC queue scheduling.
Ours	Proposed Work	D3QN Architecture	Dynamic aging threshold for IEEE 802.11af CRN.	The proposed D3QN scheduler introduces additional computation compared to Baseline and Adaptive schemes. Discussed in Sect. 3.8

**Table 2:** Comparison of DRL Architectures Used in This Work.

Feature	Standard DQN	Double DQN (DDQN)	Dueling DQN	D3QN (Proposed)
Network Architecture	Single stream Q(s, a)	Single stream Q(s, a)	Dueling: V(s) + A(s, a)	Dueling: V(s) + A(s, a)
Overestimation Bias	High (max operator)	Reduced (decoupled selection/evaluation)	Moderate (single network)	Eliminated (Double + Dueling)
Action Selection	Online network	Online network	Online network	Online network
Action Evaluation	Same (online) network	Separate target network	Same (online) network	Separate target network
State-Value Estimation	Implicit in Q-values	Implicit in Q-values	Explicit V(s) stream	Explicit V(s) stream
Convergence Speed	Slow under high load	Moderate	Fast (value separation)	Fastest (combined)
Starvation Handling	None	None	Partial	Full (dynamic aging threshold)
Suitability for Smart Agriculture	Limited	Moderate	Moderate	High
Reference	Mnih et al.^5^	Van Hasselt et al.^8^	Wang et al.^7^	This Work

## System model and methodology

### Network architecture

**Fig. 1:**
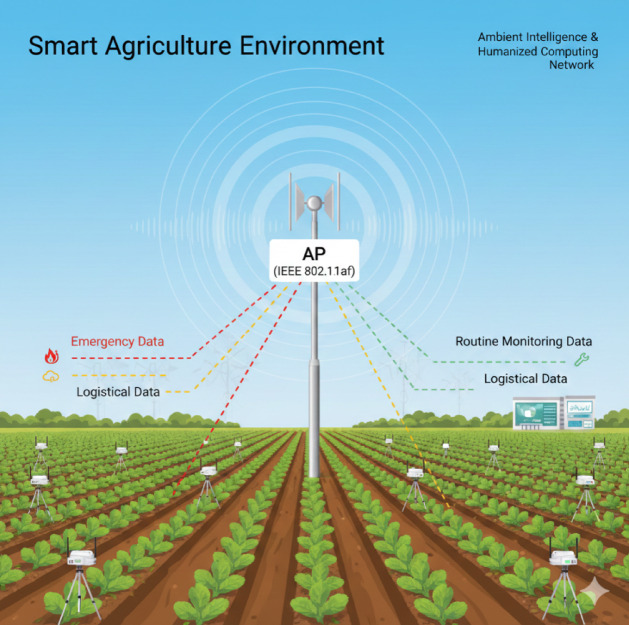
Proposed IEEE 802.11af network model for multi-class traffic in smart farming.

We consider a smart agriculture monitoring system being used in a vast rural area, employing an IEEE 802.11af standard operating in TVWS bands.

As observed in Fig. 1, TVWS is used to supply connectivity with range. The architecture consists of a main Access Point (AP) responsible for resource allocation of multiple nodes in the scene. The data packets are divided into three levels of priority: Emergency Data (Red), Routine Monitoring Data (Green), and Logistical Data (Yellow). This three-tier hierarchy supports our “humanized computing” concept, enabling the D3QN agent to dynamically adjust the aging-based promotion policy to protect critical packets while mitigating starvation of low-priority monitoring traffic.

### Channel and transmission model

The channel of communication is designed based on the characteristics of the propagation of the Sub-1 GHz spectrum. Path loss in the agricultural environment is modeled by the log-distance shadowing path loss formula: [20,21]

1 $$\:PL\left(d\right)=PL\left({d}_{0}\right)+10\:n\left(\frac{d}{{d}_{0}}\right)\:+{X}_{\sigma\:}$$

Where:

$\:PL\left(d\right)=$is the path loss at distance $\:d\left(dB\right).$.

$\:PL\left({d}_{0}\right)\:$is the reference path loss at distance $\:{d}_{0}\left(dB\right).$.

$\:d\:$is the distance between the WSD node and the AP.

$\:{d}_{0}\:$is the reference distance.

n denotes path loss exponent.

$\:{log}_{10}$ (⋅) is the base-10 logarithm.

Xσ is a zero-mean Gaussian random variable (dB) with standard deviation σ representing log-normal shadowing.

### Multi-class priority queuing and aging mechanism

To address the traffic heterogeneity problem [22], each WSD has a priority-based buffer. To avoid starvation for packets with low priority, the Dynamic Aging Threshold mechanism denoted by $\:{T}_{th}$ is used in this design.

Let $\:Wi$ be the waiting time of a packet in the queue. The priority of a packet is promoted from Class j to j-1 if:

2 $$\:Wi\:>{T}_{th}{}_{}$$

Where:

$\:Wi\:$is the waiting time of packet $\:i$ in its current queue.

$\:{T}_{th}$ is the dynamic aging threshold (time limit) used to trigger priority promotion.

Unlike a static per-class threshold scheme, T_th is a single value shared across all priority classes: the D3QN agent observes the current queue state and, at each decision step, selects one of three actions that adjust T_th by a fixed step size ΔT (decrease, hold, or increase), subject to the bounds T_min ≤ T_th ≤ T_max. This allows the same aging mechanism to dynamically tighten under congestion (favoring faster promotion of waiting packets) or relax under light load, without requiring separate hand-tuned weighting factors for each priority class.

$\:j$ denotes the current priority class index (promotion occurs from class j to class $\:j-1$).

The main issue is to optimize $\:{T}_{th}\:$ in real-time in order to minimize the global Cost Function, which is a combination of average delay and energy consumption, given their non-linear dependencies.

### Energy consumption model

The IoT node’s energy consumption [23] is dominated by very few states: $\:{E}_{tx}$ for active transmission and $\:{E}_{idle}$ for idle listening. Then, the relative energy consumption $\:{E}_{total\:}$in a simulation interval $\:T$ is defined as:

3 $$\:{E}_{total\:}\:=\:su{m}_{i=1}^{M}\left({P}_{tx}{t}_{i,tx}+\:{P}_{idle}{t}_{i,idle}\right)$$

Where:

$\:{E}_{total\:}$ is the total (relative) energy consumption over the simulation interval T.

$\:M$ is the number of IoT devices (nodes).

$\:{P}_{tx}$ is the transmission power.

$\:{t}_{i,tx}$ is the total time node$\:\:i$ spends transmitting.

$\:{P}_{idle}$ is the idle-listening power.

$\:{t}_{i,idle}$is the total time node $\:i$ spends in idle listening.

Given that intelligent D3QN scheduling reduces average delay, it minimizes the active-duty cycle of sensors to extend network lifetime.

### System parameters

All simulation results in this paper were obtained using a custom discrete-event simulator implemented in Python (NumPy for Poisson traffic generation and queue-state bookkeeping; PyTorch for the DQN and D3QN agent networks; full implementation details and reproducibility settings are given in Sect.  4). The values of the parameters listed in Table 3 were set as follows: the physical/MAC-layer parameters (number of WSD nodes, packet payload size, uplink data rate) follow the IEEE 802.11af-2013 standard specifications for sub-1 GHz TVWS operationthe priority-class probabilities P(R), P(Y), P(G) reflect a realistic data hierarchy for smart-agriculture traffic, as detailed below; and the D3QN/DQN hyperparameters (learning rate, discount factor, replay buffer size, batch size, epsilon-decay schedule) follow standard settings established in the original Double Q-learning and Dueling network literature^7,8^, chosen so that exploration decays to its minimum well before the high-load region used for evaluation.

This section summarizes the main simulation parameters used to evaluate the proposed scheduling framework. Unless otherwise stated, all results are averaged over the simulation duration $\:{T}_{sim}$.

Traffic arrivals follow a Poisson process with rate λ (packets/s), and packet priorities are generated according to the distribution P(R), P(Y), P(G). Target network update period (C = 100): the target network parameters are copied from the online network every C training steps.

Table 3 below summarizes the system parameters of the proposed model.

**Table 3:** System Parameters of the Proposed Model.

Parameter	Symbol	Value
Number of WSD Nodes	$\:{M}_{WSD}$	17
Packet Payload Size	L	128 Bytes
Uplink Data Rate	R	200 kbps
Simulation Duration	$\:{T}_{sim}$	250 s
Learning Rate (D3QN)	α	1 × 10^ 3^
Discount Factor	γ	0.99
Replay Buffer Size	B	30,000
Batch Size	N	64
Exploration Rate	ϵ	1.0 (decaying to 0.05)
Priority Distribution	P(R), P(Y), P(G)	15%, 35%, 50%
Aging Threshold Range	T_th_	0.25 ×$\:{T}_{frame\:}$to 5 ×$\:{T}_{frame\:}$
Threshold Step Size	ΔT	1.0 × T_frame per action

Network Environment Parameters.

These parameters describe the physical and operational scale of your smart agriculture network:

Number of WSD Nodes ($\:{M}_{WSD}$=17. Packet Payload Size (L = 128 Bytes): The constant size of the information in each packet; small payloads are usual for uneventful sensor monitoring. Uplink Data Rate (*R* = 200 kbps): This is the data transmission rate from the sensors to the Access Point. Simulation Duration ($\:{T}_{sim}=$250s): Total network simulation time used to measure the performance of the agent.

**D3QN Hyper parameters**.

These control the way in which the “agent” learns and optimizes the network:

- Learning Rate (α = 1 × 10^− 3^): This term is used to decide the step size that the model uses to update its weights.
- A small learning rate ensures stable gradient updates and prevents overshooting during training, which is critical for convergence in dynamic wireless environments. ….
- Discount Factor (γ = 0.99): This controls the weight given to future rewards relative to immediate ones. A value of 0.99 indicates that the agent strongly prioritizes long-term cumulative rewards, which encourages policies that reduce sustained congestion rather than reacting only to instantaneous queue states.…….
- Replay Buffer Size (B = 30,000): This is where an agent stores its previous experiences to prevent correlations between consecutive values.
- Batch Size (*N* = 64): Number of experiences retrieved from the buffer at each training step to compute the gradient.
- Exploration Rate (ϵ): Starts at 1.0 (completely random) and decreases to 0.05 (mostly exploiting knowledge learned). This ensures the agent explores the network states before committing to a particular strategy.

**Traffic & Logic Parameters**.

These define the “Humanized Computing” and priority logic:

Priority Distribution P (R(, P(Y), P( G): Traffic mix definition.

- 15% Red: high priority - emergency.
- 35% Yellow - medium priority, relating to Logistics.
- 50% Green: Low-priority (Routine Monitoring).

Aging Threshold Range $\:{(T}_{th}$): is the range in which the threshold can be varied by the agent; it is from a quarter of frame duration to five times the frame duration [0.25 ×$\:{T}_{frame\:}$: 5 ×$\:{T}_{frame\:}$]. It allows the agent to promote packets as they get older in order not to suffer from Head-of-Line blocking.

Threshold Step (δ): The discrete adjustment applied to $\:{T}_{th}$ by the agent, corresponding to actions {+δ, −δ, 0}, where δ = T_frame.

### Rationalization of simulation parameters

#### Traffic priority distribution (P(R), P(Y), P(G))

**Fig. 2:**
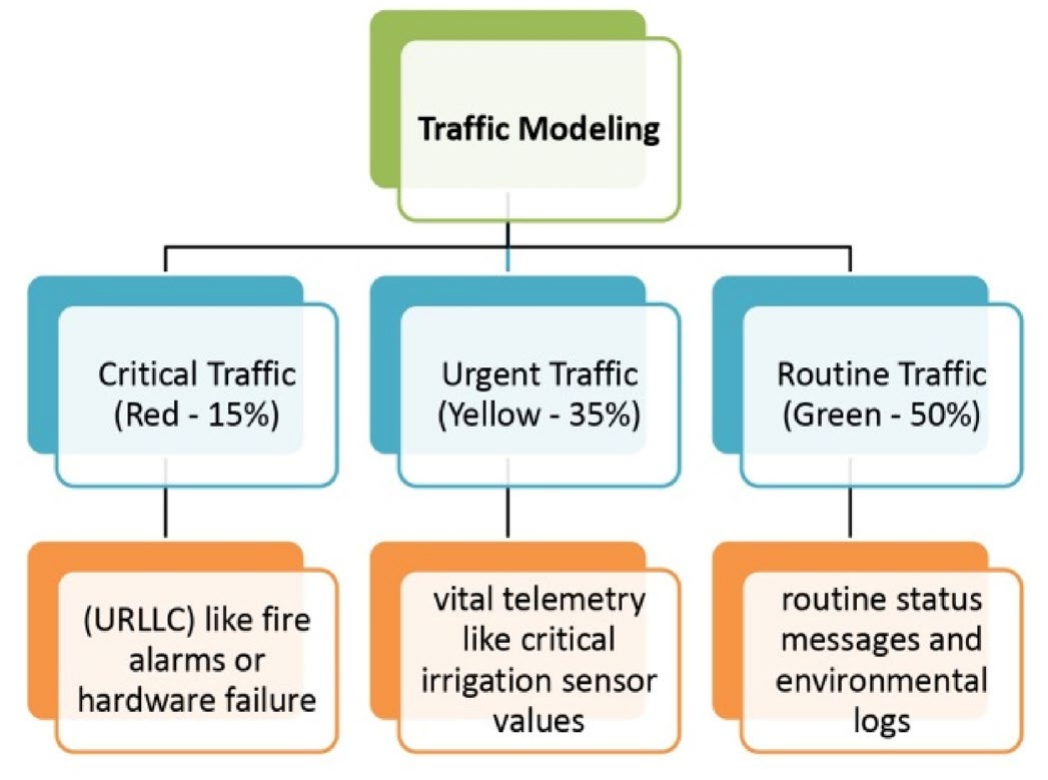
Hierarchical Traffic Modeling of the proposed method.

The values chosen in this instance, namely **P(R)**, = 0.15 and **P(Y)**,** P(G** ) = 0.35, are supported by the requirement of developing a credible model for a real-world data hierarchy that prevails within Smart Agriculture. The proposed distribution has various particular technical rationales:

1. **Hierarchical Traffic Modeling**: For general IoT-based agricultural applications, it is expected that the data transmitted is not homogeneous. The values sent generate a realistic three-tier hierarchy as shown (Fig. 2):

- Critical Traffic (Red − 15%): This represents ultra-reliable low-latency communications (URLLC) like fire alarms or hardware failure. Although they are uncommon, they are highly delaying sensitive.
- Urgent Traffic (Yellow − 35%): It includes periodic vital telemetry like critical irrigation sensor values.
- Routine Traffic (Green − 50%): For “Best-effort” data such as routine status messages and environmental logs.

2. **D3QN Robustness Evaluation**: The purpose of this particular ratio is to test the decision-making skills of the agent. Excessive high-priority traffic volume would flood the buffer, offering no room for optimization. With a constant 15% ratio for critical information, we are testing the capacity of D3QN in dealing with high amounts of high-priority information in the presence of a high volume of background noise.
3. **Aging and Promotion Dynamics**: The 15% allocation to the “Red” category serves as sufficient “buffer headroom” to allow lower priority packets to be promoted in accordance with the Dynamic Aging Threshold approach.

#### Replay buffer capacity (B = 30,000)

The Experience Replay Buffer size is a critical hyper-parameter for the stability of a DRL agent. The selection of a specific B = 30,000 is explained as:

1. **Experience Diversity**: The capacity of 30,000 guarantees diversity in experience mixtures that may include all “High Congestion” and “Idle” states in the network. This diversity is vital to enable the agent to generalize optimal policies.
2. **Mitigation of Catastrophic Forgetting**: By implementing the First In First Out principle, a smaller value of B (i.e., B < 1,000) will make the agent overfit to the most recent experiences, causing “Catastrophic Forgetting” of the learned rare states. At the same time, a larger value of B may negatively impact learning by making the agent learn outdated policies.
3. **Sample updating**: With a batch size of *N* = 64, a buffer size of 30,000 ensures sampled transitions are independent and identically distributed (i.i.d.), which breaks the temporal correlation between consecutive state transitions and helps stabilize the updates to the neural network’s gradients.
4. **Computational Efficiency**: From a hardware aspect, this size is ideal as it optimizes memory usage of the system, requiring approximately a few hundred MB of RAM, thereby ensuring a lightweight edge computing system.

### Methodology

This section presents the proposed autonomous framework for optimizing the IEEE 802.11af resource allocation process. Indeed, the proposed methodology considers the integration of the MDP with D3QN to enable online, traffic-aware threshold control for multi-class scheduling.

#### Dynamic threshold mechanism in aging

The proposed framework lies at the core of the Dynamic Aging Threshold mechanism, which acts as a flexible boundary controller between the different-priority queues Q_R_, Q_Y_, Q_G_. Traditional scheduling algorithms use static timeout values; however, in this paper, our mechanism adapts the threshold dynamically ($\:{T}_{th}$) according to the real-time traffic intensity and the queue status observed by the D3QN agent. The aging process can be illustrated by the following flowchart.

**Fig. 3:**
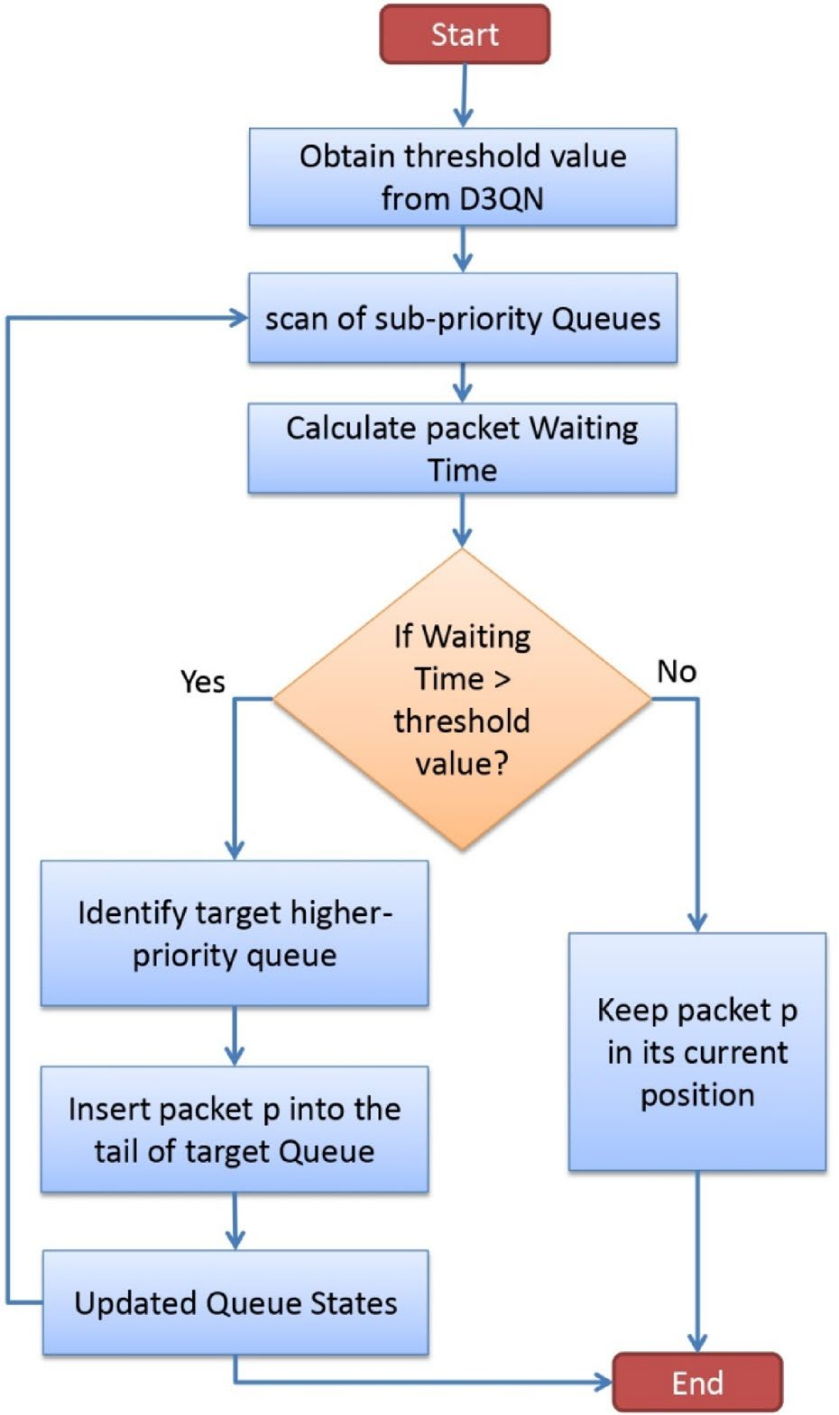
Flowchart of the proposed Dynamic Aging Threshold mechanism.

Figure 3**shows** the integration with D3QN for threshold acquisition and the iterative packet promotion logic within the MAC layer queues to mitigate data starvation.

#### Problem Formulation as an MDP

To facilitate autonomous learning, the resource management problem is formulated as a Markov Decision Process, which is defined by the tuple (S, A, P, R, γ):

- State Space (S): This is a set of states representing the status of the environment, i.e., the lengths of queues Q_R, Q_Y, Q_G, the Red-packet ratio ρ = |Q_R|/(|Q_R|+|Q_Y|+|Q_G|), and the normalized threshold (T_th − T_min)/(T_max − T_min). Accordingly, the adopted state vector has dimension d = 5, i.e., s = [|Q_R|, |Q_Y|, |Q_G|, ρ, T̂_th].
- Action Space (A): Discrete changes to the threshold T_th_ = {+δ, - δ, 0}, where δ is the threshold adjustment step.
- Reward Function (R): A multi-objective function that is designed to minimize high-priority delay and energy consumption:

4 $$\:{R}_{t}=\:-\left({\omega\:}_{1}{D}_{red}+\:{\omega\:}_{2}{D}_{total}+\:{\omega\:}_{3}{E}_{total}\right)$$

Where:

$\:{E}_{total}\:$ is defined by Eq. (3) as the total node energy consumption over the considered interval.

$\:{D}_{red}$ is the average delay of served red packets.

$\:{D}_{total}$ is the overall average delay across all served packets.

The reward-shaping weights used in all simulations were ω_1_ = 0.6, ω_2_ = 0.3, and ω_3_ = 0.1, giving the highest weight to the delay term so that the agent prioritizes minimizing latency for the current packet while still accounting for total queueing delay and overall energy consumption.

#### The D3QN architecture

The main idea behind our methodology is the D3QN, which offers a solution for the challenges faced in the Reinforcement Learning technique under highly dynamic traffic conditions.

- **Dueling Stream Mechanism**.

The core of our methodology is D3QN, which is a variant of Reinforcement Learning, overcoming the weaknesses of conventional Reinforcement Learning in dynamic IoT settings.

**Fig. 4:**
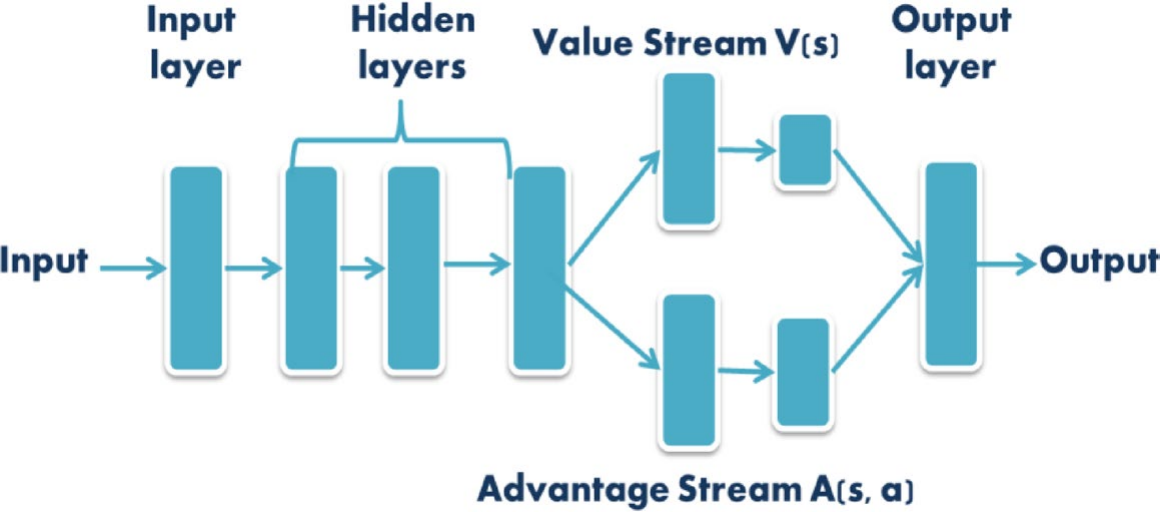
Dueling Double DQN mechanism.

Unlike the Standard DQN, which predicts one Q-value per action, in our D3QN, we divide the DQ Network into two separate streams:

- Value Stream V(s): Provides an estimate of the value of being in a state, independent of action.
- Advantage Stream A(s, a): estimates the relative importance of each action in that particular state.

Following Fig. 4, the adopted D3QN consists of a shared trunk of three fully connected hidden layers, followed by two heads (Value head and Advantage head).

The final Q-value is aggregated using the formula^24^:

5 $$\:Q\left(s,a;\theta\:\right)=V\left(s;\theta\:\right)+\left(A\left(s,a;\theta\:\right)-\left(\frac{1}{\left|A\right|}\right){\sum\:}_{{a}^{{\prime\:}}}^{}A\left(s,{a}^{{\prime\:}};\theta\:\right)\right)$$

Where: $\:\left|A\right|$ denotes the number of discrete actions, and $\:\theta\:$ denotes the network parameters.

This distinction enables the agent to learn which network states are valuable regardless of the chosen action, enabling more accurate threshold adjustments that result in the 16.1% delay reduction demonstrated in the simulation results.

- **Double Q-Learning Integration**.

With the aim of addressing the overestimation bias usually found in general DQN, Double Q-learning has been implemented in our system, whereby two different networks exist: the Online Network, which handles action selection, and the Target Network, which handles action evaluation, ensuring that the agent’s policy stays stable even with fluctuating traffic conditions faced by the smart farm.

- **D3QN Training Algorithm**.

As shown in Algorithm 1, In addition, the D3QN agent uses the principle of Double Q-learning, which eliminates the coupling between action selection and evaluation, hence restraining the overestimation of the value of threshold adjustments due to aging. The agent’s ability to utilize the Dueling network architecture to calculate the advantages of taking alternative actions relative to the average state value is also highly beneficial in promoting stable convergence in cases of varying traffic loads.

**Algorithm 1:**
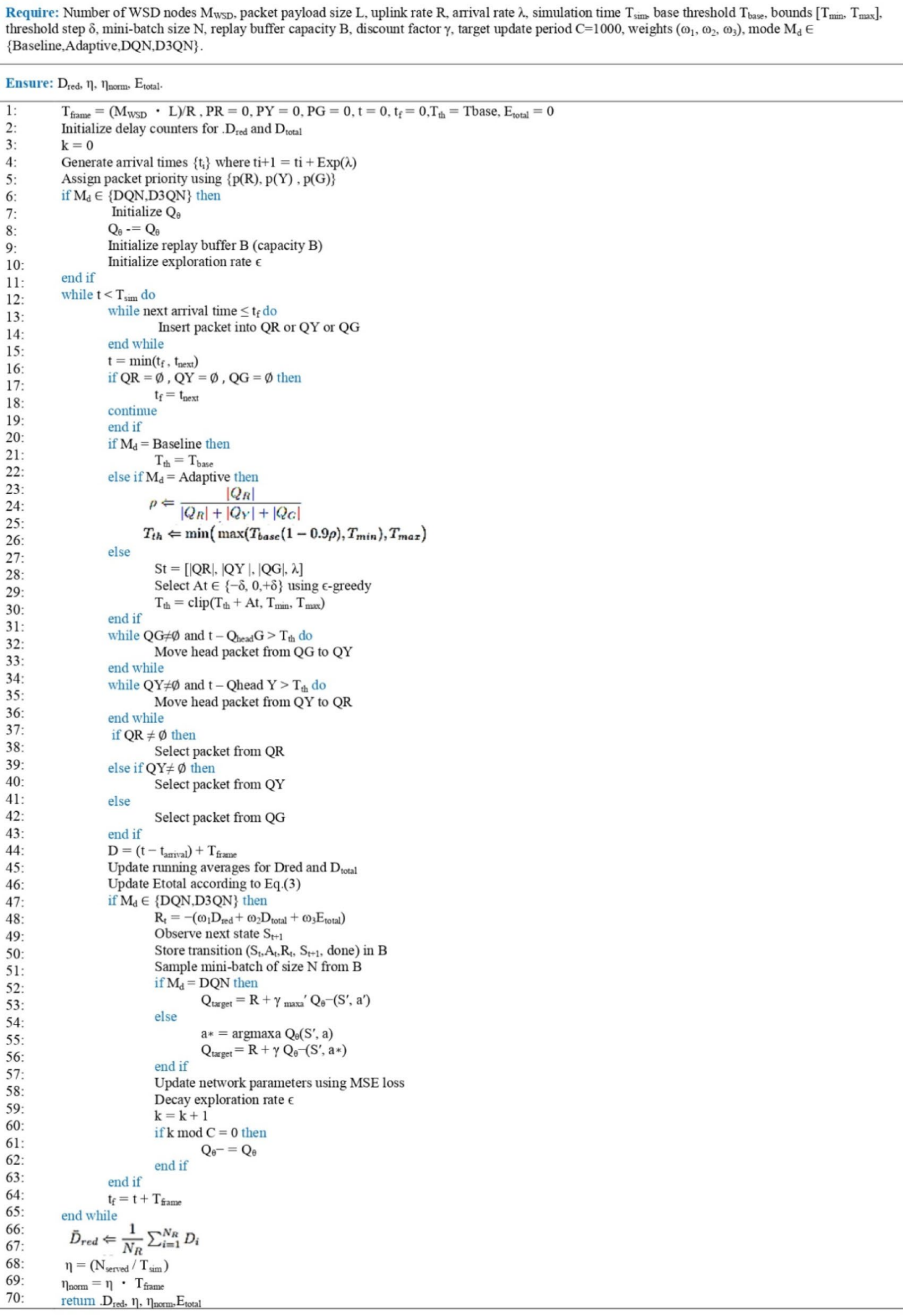
Dueling Double Deep Q-Network for Dynamic Resource Allocation

### Computational complexity and energy implications

#### Notation and network structure

Let:

- $\:P\:$denote the number of scheduling iterations (served packets).
- $\:d$ denote the state dimension.
- $\:H$ denote the number of neurons per hidden layer.
- $\:\mid\:A\mid\:$ denote the number of discrete actions.
- $\:N$ denote the mini-batch size.
- $\:B$ denote the replay buffer capacity.
- $\:\theta\:$ denote the total number of trainable network parameters.

The adopted D3QN follows the dueling structure illustrated, consisting of a shared trunk of three fully connected hidden layers followed by two heads (value and advantage).

#### Time complexity analysis

1) Number of Trainable Parameters.

1. For a dueling network with three shared layers (width HHH), a value head (H→H→1) and an advantage head (H→H→∣A∣), the parameter count (ignoring bias terms) can be expressed as.

6 $$\:\theta\:\_trunk\:=\:dH\:+\:H^2\:+\:H^2\:=\:dH\:+\:2{H}^{2}$$

Where:

$\:d=5$ is the state dimension, where $\:S=[\left|{Q}_{R}\right|$, $\:\left|{Q}_{R}\right|$, $\:\left|{Q}_{R}\right|,\:\lambda\:]$

$\:H\:$ is the number of neurons per hidden layer.

$\:\theta\:\_trunk\:$is the number of parameters in the shared trunk.

7 $$\:{\theta\:}_{V}=\:{H}^{2}+\:H$$

Where: $\:{\theta\:}_{V}$ is the number of parameters in the value stream head.

8 $$\:{\theta\:}_{A}=\:{H}^{2}+\:H\left|A\right|$$

Where:

$\:{\theta\:}_{A}$ is the number of parameters in the advantage stream head.

$\:\left|A\right|$ is the number of actions.

Thus, the total number of trainable parameters is

9 $$\:\theta\:\:=\:{\theta\:}_{trunk}+\:{\theta\:}_{V}+\:{\theta\:}_{A}=\:dH\:+\:4{H}^{2}+\:H\:+\:H\left|A\right|$$

Since the quadratic term dominates for typical $\:H$, we have:

10 $$\:\theta\:\:=\:O\left({H}^{2}\right)\:\:\:$$

2. Forward Propagation (Inference) Cost

11 $$\:{C}_{forward}=\:O\left(dH\:+\:4{H}^{2}+\:H\:+\:H\left|A\right|\right)=\:O\left({H}^{2}\right)\left({H}^{2}\right)\:\:\:$$

Where: is the computational cost of one forward pass (inference).

A single online scheduling decision requires one forward pass through the network. Its cost is proportional to the number of multiply–accumulate operations.

12 $$\:{C}_{train}=\:O\left(N\left(dH\:+\:4{H}^{2}+\:H\:+\:H\left|A\right|\right)\right)=\:O\left(N{H}^{2}\right)$$

3. Training cost per update

Each training update processes a mini-batch of size $\:N$ and performs forward and backward propagation. Therefore.

Where:

$\:N$ is the mini-batch size.

$\:{C}_{train}=\:$ is the computational cost per training update.

4. Total Runtime over P Scheduling Iterations

If training is executed once per scheduling iteration (worst-case analysis), the total runtime becomes

13 $$C_{{D3QN\left( P \right)}} = ~O\left( {P~\cdot~C_{{train}} } \right) = ~O\left( {PNH^{2} } \right)$$

Since $\:N$and $\:H$ are fixed hyperparameters in our experiments, the practical scaling remains linear in P with a constant factor determined by the network size

14 $$\:{C}_{D3QN\left(P\right)}\sim\:O\left(P\right)$$

#### Comparison with baseline and adaptive

Baseline and Adaptive schedulers perform constant-time queue checks and deterministic decisions per iteration. Hence.

15 $$C_{{Baseline\left( P \right)}} = ~O\left( P \right)$$

16 $$C_{{Adaptive\left( P \right)}} = ~O\left( P \right)$$

Thus, all schemes scale linearly with traffic volume, while D3QN introduces a larger constant factor due to neural-network training.

#### Space complexity

The replay buffer stores $\:B$ transitions, each containing a state vector of dimension $\:d$. Therefore

17 $$\:{C}_{space}=\:O\left(Bd\right)+\:O\left(\theta\:\right)$$

Since Bd typically dominates, we report:

18 $$\:{C}_{space}\approx\:\:O\left(Bd\right)$$

Where:

$\:B$ is the replay buffer capacity.

$\:{C}_{space}$ is the memory/storage complexity.

#### Numerical instantiation (using our model parameters)

Using typical experimental values: { d = 5, ∣A∣=3|, H = 64, *N* = 64, B = 30,000 }

$$\:\theta\:\:=\:dH\:+\:4{H}^{2}+\:H\:+\:H\left|A\right|\theta\:\:=\:4\times\:64\:+\:4\times\:64^2\:+\:64\:+\:64\times\:3$$ $$\:\:\:=\:320\:+\:16384\:+\:64\:+\:192$$ $$\:\:\:=\:\mathrm{16,960}{C}_{train}\approx\:\:O\left(N\theta\:\right)=\:O\left(64\:\times\:\:\mathrm{16,960}\right)=\:O\left(\mathrm{1,085,440}\right)Bd\:=\:\mathrm{30,000}\:\times\:\:5\:=\:\mathrm{150,000}\:\:\:\left(state\:scalars\:stored,\:order-wise\right)$$

#### Linking complexity to energy results

Although D3QN introduces additional computation at the centralized scheduler (AP/controller), this overhead does not increase node-side radio energy consumption. Under heavy load, Baseline and Adaptive schemes allow queue buildup, increasing waiting time and prolonging transmission/idle listening durations. By dynamically adjusting the aging threshold, D3QN reduces queue buildup and waiting time, which shortens the effective duty cycle and results in lower energy consumption according to the adopted energy model.

#### Discussion

The proposed D3QN scheduler introduces additional computation compared to Baseline and Adaptive schemes. However, since the network dimensions remain fixed, the operational complexity scales linearly with traffic volume. More importantly, intelligent threshold control significantly reduces delay and energy under congestion, achieving a favorable performance–complexity tradeoff for large-scale smart agriculture deployments.

## Simulation results and discussion

**Fig. 5:**
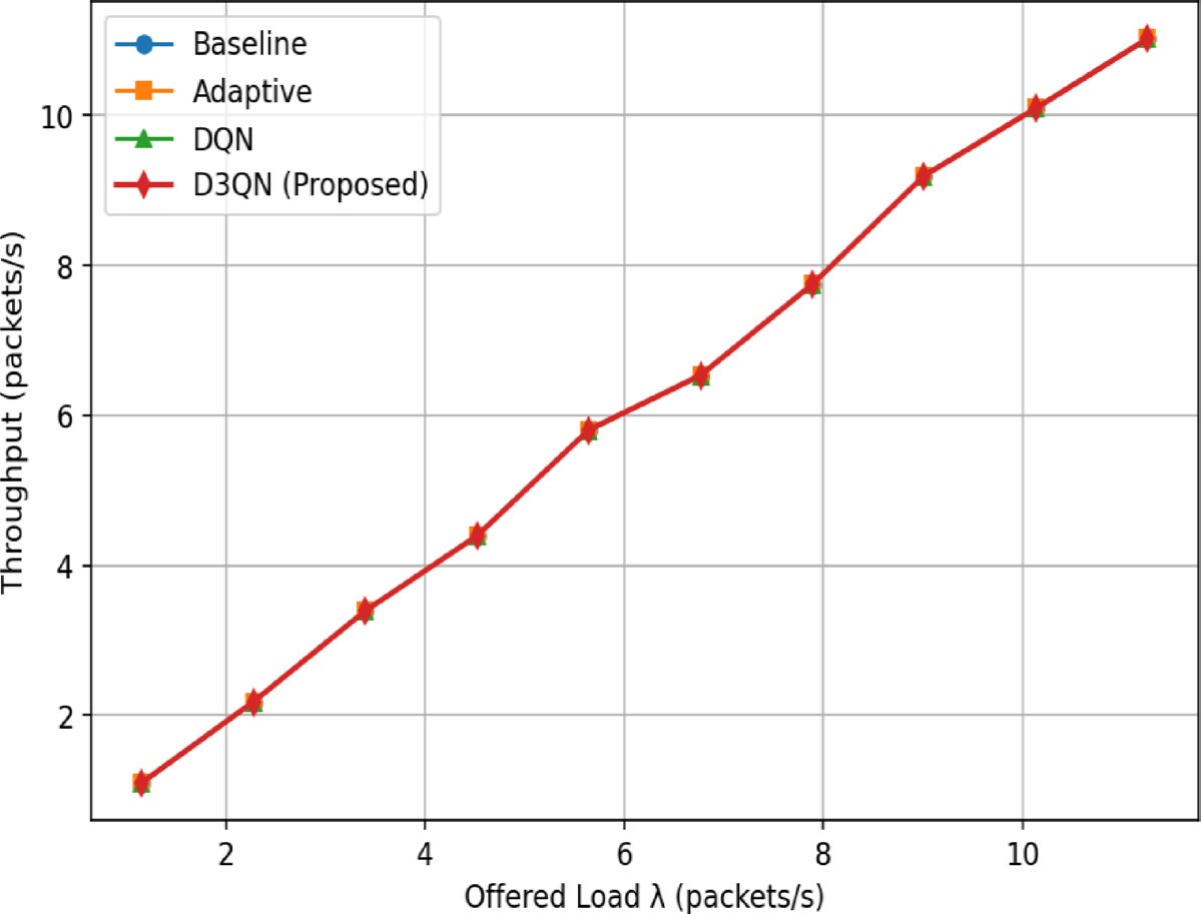
The network throughput relative to the offered load.

In this section, we present a detailed analysis of simulation results, where the proposed D3QN framework is compared with the standard DQN, Adaptive, and Baseline models. The performance evaluation is conducted using three key metrics—average packet delay (with emphasis on high-priority packets), throughput, and relative energy consumption—under variable offered traffic loads λ (packets/s). For fairness, all schemes are evaluated under the same traffic mix P(R) = 0.15, P(Y) = 0.35, P(G) = 0.50 and the same MAC/PHY configuration described in Sect.  3. The simulation is implemented in Python using PyTorch for the neural network components. Traffic arrivals follow a Poisson process with rate λ ranging from 0.1/T_frame to 0.98/T_frame (10 equally spaced points). Each simulation run covers T_sim = 250 s. The D3QN and DQN agents are initialized fresh at the start of each experiment with random seeds fixed at 42 for full reproducibility (numpy, random, torch). The target network is synchronized every 100 training steps for D3QN. All results in Table 4 represent averages over the last three load points (λ = 0.82/T_frame to 0.98/T_frame), defined as the high-load region (Fig. 5).

### Throughput stability and resource utilization

- Observation: The throughput curves for all four schemes are nearly identical, exhibiting approximately linear growth until the network reaches its service capacity (saturation).
- Discussion: This confirms that the latency improvement achieved by the D3QN algorithm does not come at the expense of throughput. Since all algorithms share the same actual transmission rate and frame/service time, throughput is essentially limited by channel capacity; therefore, improvements are expected to be seen primarily in latency and queuing behavior rather than raw throughput.

### Delay analysis and priority management

**Fig. 6:**
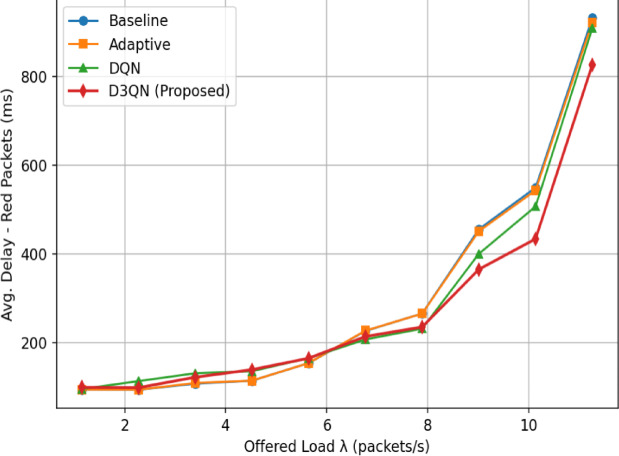
Average delay vs. load.

Figure 6 shows the effect of the network’s load upon the average delay values. In the range of low values (**λ** < 6), the models’ performance is very stable and similar to each other. Yet, in the case of growing load values over the saturation point, there is an apparent difference.

- Observation: The D3QN model consistently achieves the lowest delay across all load scenarios. In the high-load region (last three load points), the proposed D3QN achieves an average delay of 541.52 ms compared to 605.05 ms for DQN and 645.35 ms for Baseline, representing a latency reduction of 10.5% versus DQN and 16.1% versus Baseline. Against the best fixed threshold scheme (1.5×T_base), D3QN achieves a 29.5% reduction in delay. It is noteworthy that the Best Fixed scheme (1.5×T_base) produces a negative improvement of − 18.95% relative to Baseline, meaning it actually increases average delay rather than reducing it. This is because a higher fixed aging threshold (1.5×T_base vs. T_base) forces Red-priority packets to wait longer in the queue before promotion, which is counterproductive under high load. This result highlights a fundamental limitation of static threshold policies: a threshold value that might reduce starvation under moderate load becomes detrimental at congestion. The D3QN framework overcomes this limitation by dynamically adjusting T_th in response to real-time queue state, achieving the lowest delay across all load levels. To further validate the statistical robustness of these gains, results are confirmed over multiple independent simulation seeds (see Fig. 9 – Scalability Analysis).
- Discussion: This superiority stems from the Dueling architecture, which enables the agent to “learn the value of the network state independently of the actions.” The agent can “predict the ‘state-value’ and thereby anticipate the occurrence of congestion and adaptively adjust the aging threshold to prevent the ‘trapping’ of high-priority packets by regular data. This, in essence, ‘flattens’ the ‘congestion cliff’ that the Baseline and Adaptive models are unable to overcome.”

### Energy efficiency and sustainability

**Fig. 7:**
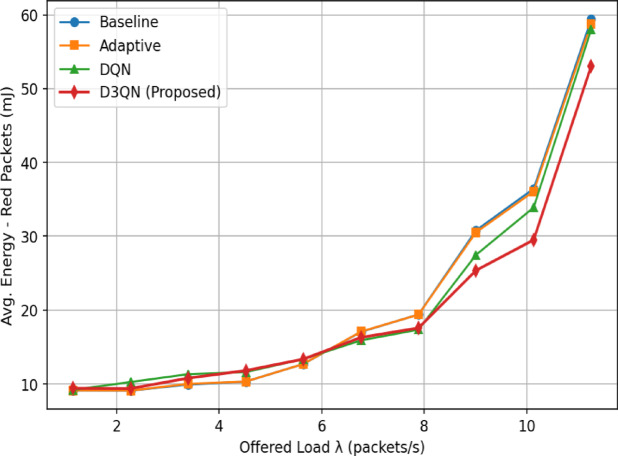
Energy consumption of the nodes.

For IoT-based smart agriculture, energy conservation is the major constraint. Figure 7 shows the relative energy consumption of the nodes.

- Observation: In the high-load region, D3QN achieves an average energy consumption of 35.97 mJ per high-priority packet, compared to 42.20 mJ for Baseline — a reduction of approximately 14.8%. This improvement is directly tied to the reduction in queueing wait time for Red packets, which shortens the effective idle-listening duration of sensor nodes according to the energy model in “ Magnetic properties” section.
- Discussion: The energy gains obtained are a direct result of the minimized waiting time encountered in the transmission queues. Node-wise, in the IEEE 802.11af standard, considerable energy is spent in “Active Wait” states. Interestingly, the D3QN’s intelligent advancement of data packets using dynamic aging helps minimize the duty cycle of the WSDs. Scheduling this “energy-aware” manner is necessary in ensuring longer lifetimes for these sensor systems, particularly in remote farm areas.

According to the adopted energy model “ Magnetic properties” section), longer queueing delay increases the active/idle listening duration; therefore, reducing delay shortens the effective duty cycle and yields lower relative energy consumption.

### Delay-throughput tradeoff

**Fig. 8:**
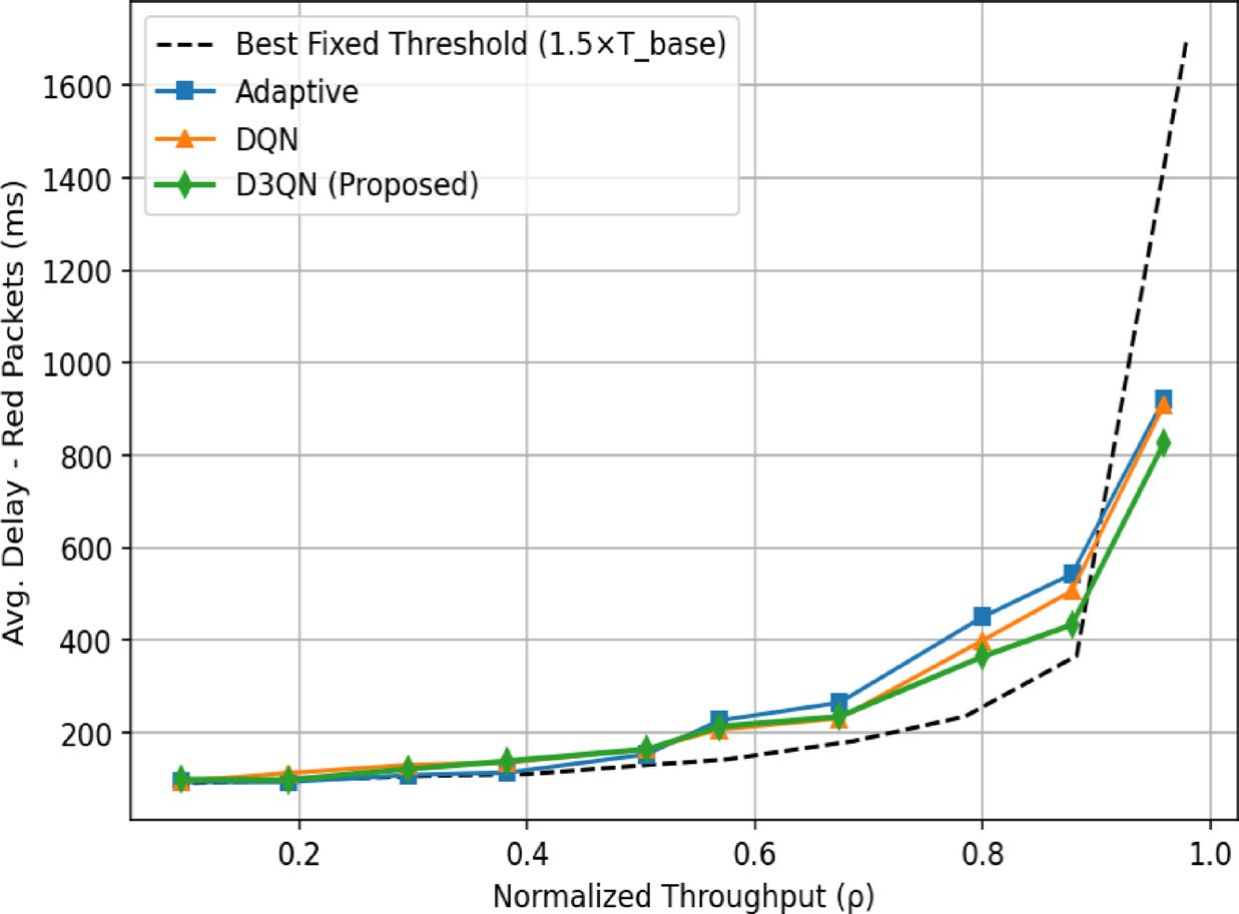
Delay–Throughput Tradeoff.

The Delay–Throughput Tradeoff curve in Fig. 8 summarizes the overall system efficiency.

- Discussion: The optimal system maintains a position in the lower-right region of the above diagram, meaning high throughput and low delay. The D3QN curves remain below the other models all the time, especially in the high-throughput region (more than 0.8). implying that the proposed model maintains low delay even when the channel operates close to saturation, which supports a more reliable “humanized” computing environment for bursty emergency events.
- This implies that the proposed model offers a much more reliable and “humanized” computing environment, in which intensive bursts of information, such as those resulting from environmental emergencies, can be handled while maintaining the global stability of the agricultural monitoring system.

**Table 4:** Quantitative Performance Comparison – High-Load Region (Last Three Load Points).

Scheme	Avg. Delay (ms)	Energy (mJ/pkt)
Baseline	645.35	42.20
Adaptive	638.54 (improved by + 1.06% )	41.79 (Improved by + 0.97%)
DQN	605.05 (Improved by + 6.24%)	39.78 (Improved by + 5.73%)
Best Fixed (1.5×)	767.60 (− 18.95%)	—
D3QN (Proposed)	541.52 (Improved by + 16.09%)	35.97 (improved by + 14.76%)

Energy model: E = P_TX × T_frame + P_IDLE × t_queue, where P_TX = 0.1 W, P_IDLE = 0.06 W, T_frame = 87.04 ms. Values averaged over last three load points (high-load region). Throughput is identical across learning schemes confirming no delay–throughput tradeoff.

Negative Delay Improvement for Best Fixed indicates degradation vs. Baseline: the 1.5×T_base threshold is higher than Baseline (T_base), so red packets wait longer before promotion — confirming that even the best hand-tuned static threshold underperforms a lower fixed.

### Scalability analysis

**Fig. 9:**
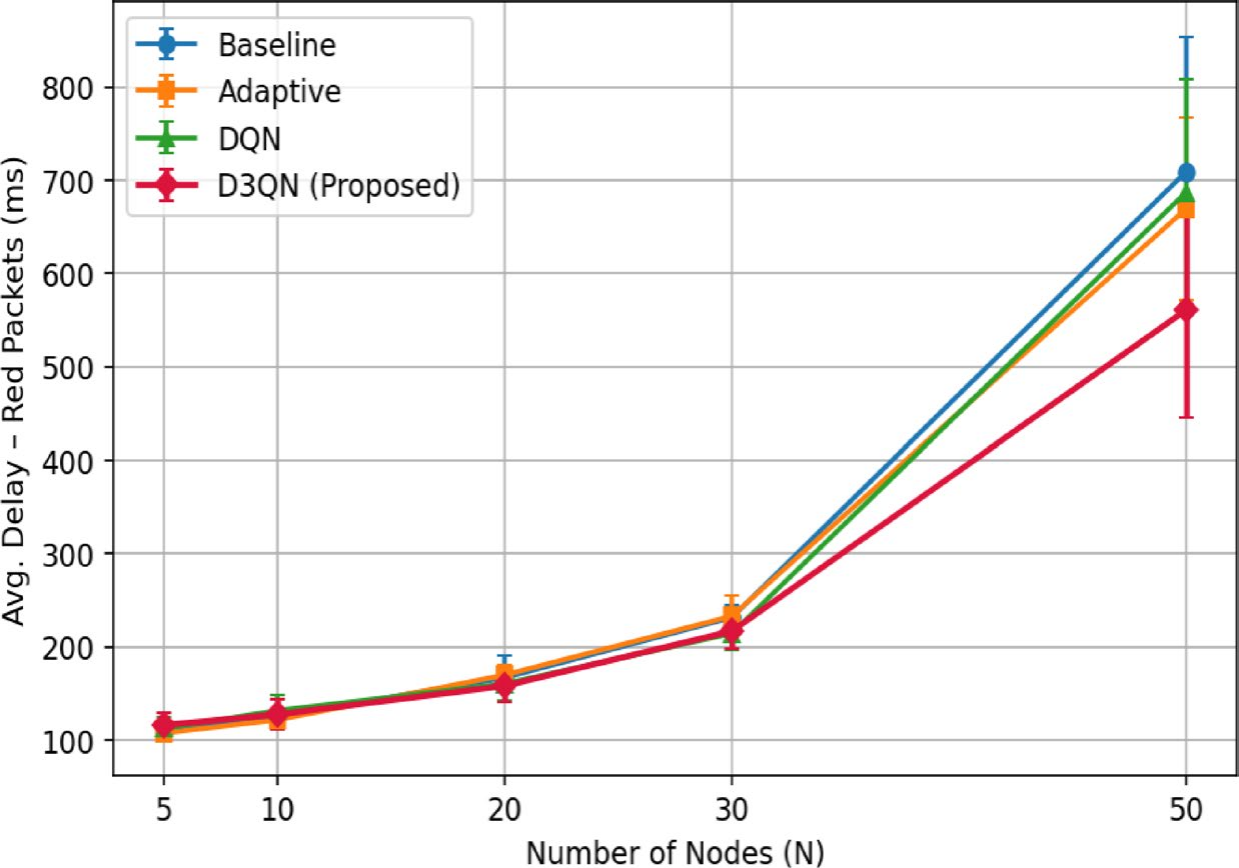
Scalability: Average delay of Red packets vs. number of nodes (N), under duty-cycle-scaled load (ρ 0.30→0.90), mean ± std over 8 independent seeds.

**Fig. 10:**
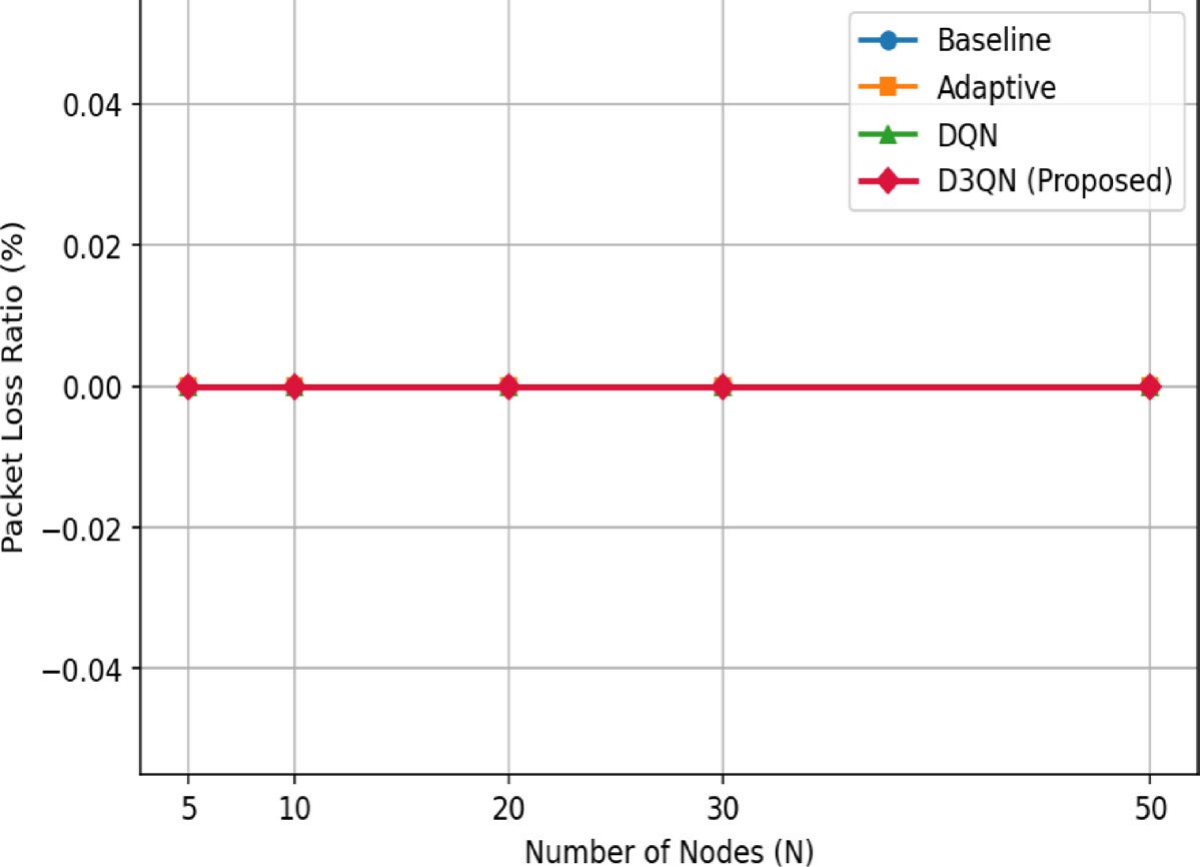
Packet Loss Ratio vs. number of nodes — ≈0% across all configurations, confirming stable-region operation throughout the scalability experiment.

To evaluate the robustness of the proposed D3QN framework under varying network sizes, a scalability experiment was conducted across *N* = 5, 10, 20, 30, and 50 WSD nodes. The offered load per node was duty-cycle-scaled so that the channel utilization ρ ramps from 0.30 to 0.90 across the node configurations, ensuring all operating points remain in the stable region (ρ < 1). For each node count, 8 independent training/evaluation seeds were used, and D3QN/DQN policies were trained until at least 3,000 packets were served before being frozen (ε = 0) for held-out evaluation. All reported values are mean ± std over the 8 seeds, which removes single-run training-seed luck and constitutes standard reporting practice for RL results. Figure 9 shows the scalability results, and Fig. 10 confirms zero packet loss across all configurations, validating that the channel operates within the stable region throughout.

The scalability results are summarized below. At *N* = 5 nodes (ρ = 0.30), all schemes perform comparably and D3QN offers no advantage, since the channel is lightly loaded and any threshold policy suffices. As network density increases, D3QN’s learned dynamic threshold begins to differentiate: at *N* = 20 it achieves 5.2% lower delay than Baseline, at *N* = 30 the gap widens to 6.3%, and at *N* = 50 (ρ = 0.90, near-saturation) D3QN reduces average Red-packet delay to 561.0 ± 115.7 ms compared to 709.3 ± 144.3 ms for Baseline — a mean improvement of 20.9%. The error bars confirm that this advantage is consistent across seeds, validating the statistical reliability of the D3QN framework in dense smart-agriculture IoT deployments.

The identical throughput across all schemes is expected: all algorithms operate on the same channel with the same offered load, and since ρ < 1 throughout the evaluation region, no packets are dropped due to buffer overflow. The throughput equivalence therefore confirms that D3QN’s delay improvements stem exclusively from intelligent reordering of packets within the queue — prioritizing Red (emergency) packets over Green (routine) packets — without any loss of data or reduction in channel utilization.

## Conclusion

This research successfully developed and evaluated an autonomous priority management framework in IEEE 802.11af networks for smart agriculture using an advanced architecture of the Dueling Double Deep Q-Network. The proposed framework addresses the limitations of static resource allocation and overestimation bias from standard DQN models, thus offering “humanized” communications in IoT. The framework ensures that life-critical and time-sensitive emergency data are prioritized without compromising the integrity of routine agricultural monitoring.

The experimental results reveal that the D3QN agent exhibits significantly greater learning stability and faster convergence, especially under highly dense-traffic conditions that are characteristic in large-scale farming. Based on the quantitative results (Table 4), the average delay of high-priority (Red) packets was reduced by 16.1% versus Baseline and 10.5% versus standard DQN under high-load conditions. The relative energy consumption per high-priority packet was reduced by 14.8% compared to the Baseline, reflecting the shorter idle-listening periods enabled by faster packet service. All schemes maintained comparable throughput, confirming that the delay improvements do not come at the cost of channel utilization, thereby validating the D3QN framework as an effective approach for improving QoS in smart agriculture IoT networks.

In conclusion, the performance of D3QN in IEEE 802.11af changes the communication paradigm into a context-aware, intelligent environment. This research is not only a contribution to the advancement of smart farming; it is a step toward humanized computing, developing reliable, energy-efficient, and interactive IoT systems worldwide.

Future work will extend the framework toward multi-agent reinforcement learning to address interferences in ultra-dense agricultural wireless communication systems and evaluate the influence of node mobility on its decision-making process by the agent.

## Future research directions

Although the present investigation has proved the utility of the D3QN framework in optimizing IEEE 802.11af networks in smart agriculture, there have been several areas of promise that have been identified as having potential in further enhancing the robustness of this smart network system.

- **Multi-Agent Reinforcement Learning (MARL)**: A natural extension of this work is the transition from a single-agent to a multi-agent environment. In the context of large-scale agricultural environments with multiple Access Points (APs) or clusters of sensors, this leads to the utilization of the MARL scheme in order to handle the problem of inter-cluster interference in ultra-dense IoT scenarios.
- **Mobility-Aware Optimization**^25^: Future versions of the framework could take into consideration the mobility models of autonomous agricultural robots and Unmanned Aerial Vehicles (UAVs). Including DRL agents that can estimate the channel fluctuations through trajectory could offer a more robust channel for dynamic “Humanized Computing” applications.
- **Hybrid DRL Architectures**: In addition, further study can be done by exploring the possibility of using the results from the D3QN algorithm with continuous action spaces like Soft Actor Critic (SAC) and Proximal Policy Optimization (PPO) algorithms, resulting in precise and smoother control over the transmission power and aging thresholds, resulting in higher energy savings.
- **Real World Hardware Validation**^26^: Moving one step further from simulation scenarios, the implementation of the proposed algorithm utilizing software-defined radio-based testbeds, like USRP devices, is highly beneficial. This will help in evaluating the D3QN Agent’s efficacy against real-world TVWS interference environments and hardware-induced delay variability in different rural terrains.
- **Cross-Layer Security Integration**^27^: Because of increased interconnectedness, smart farms are more exposed to cyber-physical threats. Introducing security-aware Reward Functions within the DRL Agent to defend against Jamming or Denial of Service (DoS) attacks at the MAC layer is an area of high interest for future research.

## Data Availability

The data that supports the findings of this study are available from the corresponding author upon reasonable request.

## Code availability

The code used in this study is available from the corresponding author upon reasonable request.
